# A Cognitive Neuroscience View of Schizophrenic Symptoms: Abnormal Activation of a System for Social Perception and Communication

**DOI:** 10.1007/s11682-008-9052-1

**Published:** 2009-03-01

**Authors:** Cynthia G. Wible, Alexander P. Preus, Ryuichiro Hashimoto

**Affiliations:** Department of Psychiatry, Harvard Medical School, Boston, MA, USA; Department of Psychiatry, Brockton VA Medical Center, Brockton, MA, USA; Department of Psychiatry, Harvard Medical School, Boston, MA, USA; Department of Psychiatry, Harvard Medical School, Boston, MA, USA; Department of Psychiatry, Brockton VA Medical Center, Brockton, MA, USA

**Keywords:** Schizophrenia, Temporal lobe, Superior temporal sulcus, Inferior parietal, Temporo-parietal junction, Hippocampus, Language, Default system

## Abstract

We will review converging evidence that language related symptoms of the schizophrenic syndrome such as auditory verbal hallucinations arise at least in part from processing abnormalities in posterior language regions. These language regions are either adjacent to or overlapping with regions in the (posterior) temporal cortex and temporo-parietal occipital junction that are part of a system for processing social cognition, emotion, and self representation or agency. The inferior parietal and posterior superior temporal regions contain multi-modal representational systems that may also provide rapid feedback and feed-forward activation to unimodal regions such as auditory cortex. We propose that the over-activation of these regions could not only result in erroneous activation of semantic and speech (auditory word) representations, resulting in thought disorder and voice hallucinations, but could also result in many of the other symptoms of schizophrenia. These regions are also part of the so-called “default network”, a network of regions that are normally active; and their activity is also correlated with activity within the hippocampal system.

## Review

### Introduction

Auditory verbal hallucinations (AVH) are a hallmark symptom of schizophrenia; up to 74% of patients experience them (Silbersweig and Stern 1996). To quote Bleuler (Bleuler 1950), “Almost every schizophrenic who is hospitalized hears “voices,” occasionally or continually”. He also noted that the hallucinations are usually in the form of voices or speech, not music or other auditory percepts, and are much more common than hallucinations in other modalities. Verbal learning and memory have also been found to be disproportionately impaired in schizophrenia (Heinrichs and Zakzanis 1998, Saykin et al. 1994). Schizophrenic symptoms are very diverse, and in addition to language related symptoms, include many seemingly disparate symptoms such as social withdrawal, visual hallucinations of people, somatic delusions, delusional jealousy and persecutory delusions (to name a few). For a brief descriptive overview of symptoms, see “Appendix 1”. The diverse range of modalities and representational systems involved seem to preclude a simple explanation, and therefore most current theories of schizophrenia involve abnormal executive control or feedback from frontal lobe to posterior regions (Goldman-Rakic 1991).

The fact that auditory hallucinations are a prominent symptom and usually consist of voices suggests to us that the language system is abnormal in schizophrenia. The language system (speech perception and production) only exists in humans and therefore is relatively difficult to study with neuroscience techniques. Hallucinatory syndromes also exist in the visual system which, unlike the language system, has been well characterized in non-human primates. The visual system is organized as maps of external space, with each map representing different visual attributes such as color, form and movement. In (non-psychotic) patients with visual hallucinations due to damage in the visual system or blindness, the form of the hallucination matched the functional processing of the cortical module(s) activated during the hallucination. Color hallucinations were associated with activity in color regions, hallucinations of faces with activity in face regions, and hallucinations of objects with activity in regions representing visual objects (Ffytche et al. 1998, Santhouse et al. 2000). Hallucinations resulted in increased activation of the particular associated brain region during the experience and there was also a general increase in the baseline of activity of the affected regions between hallucinations (Ffytche et al. 1998, Santhouse et al. 2000). If abnormal activity in one functional region of cortex extends into an adjacent region, then the components of function for each separate area can contribute, creating a hallucinatory syndrome (Santhouse et al. 2000). Brain damage can also result in high-level, conceptually organized hallucinations such as seeing written text sentences (Ffytche et al. 2004). A (nonpsychotic) patient with a posterior temporal/parietal occipital lesion presented with text hallucinations that were in the form of commands and sentences (sometimes with replies).

We propose that schizophrenic symptoms also arise from over-activation of cortical regions whose representational content is closely related to the symptom. As with visual hallucinations, the activation of one or more cortical modules during the experience of a symptom (i.e. hallucination), could also extend to closely related additional cortical regions, resulting in additional symptoms. We will examine findings from cognitive neuroscience to show that many of the diverse symptoms of schizophrenia could stem from abnormal activity in a system that may be used for dynamic social perception, communication and reaction; much of the evidence that we discuss will come from cognitive studies of healthy subjects. Classical studies of language function focus on concepts such as phonemes, semantic representations and syntactic processing. Within and near the territory of the (posterior) language regions that subserve these functions and that are activated by language perception and production tasks, there are also systems for processing social cognition and self representation. Investigations of these systems focus on concepts such as attribution, intention, agency, and emotion. These cognitive neuroscience domains come together under the rubric of social communication, and these functions are used during conversation with another person. During conversation, speech (voices) are heard and produced. At a phenomenological level, conversation also involves the perception, production and interpretation of gestures (of the body and face) and the shifting or control of attention. Gestures and speech can also convey emotional information that is used to perceive and react within a conversation and that can impart meaning or context for linguistic representations. Conversation with another person also involves representational systems for individuals (differentiation between the self and other) and necessitates the representation and working memory of both the speech and gestures produced and of the point of view of both individuals (self and other), in addition to semantic and syntactic context. These linguistic and social cognitive representations are activated during conversation in a dynamic way.

At a phenomenological level, it is easy to see how these representational systems could come into play during conversation and that there is a conceptual overlap between social cognition and speech processing. More formal theoretical conceptions of speech processing also include aspects of social cognition and in parallel, evidence from systems level neuroscience and cognitive neuroscience shows overlap between speech processing and social cognition. Recent theoretical formulations of voice processing incorporate not only linguistic representation (e.g. physical aspects of speech such as formant transitions, syllabification, etc.), but include the fact that the voice signal contains other information about individuals (person identification, age, body size, etc.) and emotional content (Belin et al. 2002, Campanella and Belin 2007). It has also been hypothesized that non-verbal skills such as initiating and responding to joint attention and making gestures form the basis for language learning in children (Redcay 2008); that the gesture perception and production (mirror) system in animals formed the basis for the language system in humans (Rizzolatti and Arbib 1998) and that language or speech can be conceptualized as social skill just as following other's gaze, pointing, and gesturing (Carpenter et al. 1998, Goldin-Meadow 1998, Kreifelts et al. 2007, Redcay 2008).

There is increasing neuro-scientific evidence that the systems for processing speech and social perception (e.g. communicative face/body gestures, emotional gestures, self versus other representation and agency, the representation of other's point of view) are overlapping and/or adjacent in the brain and act as a coordinated system (Decety and Lamm 2007, Kreifelts et al. 2007). Within the current review, we will show evidence for the overlap and interrelationships between these functions in order to build the foundations for understanding the relationship between processing in this “social communication” system, voice processing and schizophrenic symptoms.

These systems for processing speech and social perception overlap within the posterior temporal, superior temporal sulcus (STS) inferior parietal (IP) regions; and these two regions are within the temporal-occipital-parietal junction (TPJ). Evidence for the neural basis of this system comes from a number of techniques, including single unit recording (extracellular neuronal recording), neuroimaging, lesion, and brain stimulation studies in both animals and humans. At the systems neuroscience level of analysis, it has been shown that portions of the TPJ contain trimodal (auditory, visual and somatosensory) and bi-modal representational regions that also provide rapid feedback and/or feed-forward input to unimodal sensory regions, and especially to the auditory cortex in animals and humans (Matsuhashi et al. 2004); see Driver and Noesselt (2008) for a review. Traditional unimodal regions can be influenced by information from other modalities with very short latencies (30 milliseconds after stimulus onset). These findings show that multimodal and convergent contextual information from visual and tactile domains is processed within auditory cortical regions and modulates the processing of auditory information. Hence, the speech and social perception (and reaction or production) systems not only overlap within the TPJ, but multimodal information from this region can also modulate the processing of auditory (or visual/somatosensory) information at very early stages of processing.

We will refer to this set of regions as a system for social communication as a short-hand term to emphasize the aspects of function that we believe are important for understanding schizophrenia. We will pull together evidence to show that a major role of these regions is in the moment to moment or dynamic processing of social situations in the service of social perception, communication, and reaction. We assert that the diverse set of symptoms that make up the schizophrenic syndrome may be produced as a result of abnormal activity in these superior temporal and inferior parietal or TPJ regions in conjunction with the hippocampal system.

Any systems neuroscience theory of schizophrenia must account for the prominence of language related symptoms (e.g. AVH) and the particular constellation of symptoms that make up the syndrome. The theory must also eventually account for cognitive deficits and also provide an explanation for why particular brain regions (implicated in the theoretical view) are vulnerable in schizophrenia. We will address these points throughout the review. We will first discuss the regions subserving language and voice perception as an entry into a more comprehensive understanding of posterior language related cortex and its relationship to schizophrenic symptoms. We will then review evidence that regions overlapping with, and/or adjacent to, the language and voice regions are involved in social cognition, emotion, the representation of the self and self/other distinction. Some of the functions of these regions include: auditory verbal working memory and the computation of semantic context; the representation and processing of facial speech and emotional gestures; familiar faces; voices; intention; attention; body gestures; the perception and integration of embodiment and extra-personal space; and the perception of autonomy and control of the self. The reader is also referred to a recent meta-analysis of functional imaging studies of the TPJ showing that the systems for attention or re-orienting, empathy, theory of mind and agency overlap within this region (Decety and Lamm 2007). Studies of non-psychotic neurologically impaired individuals or presurgical brain stimulation show that stimulation or lesions in these regions can cause many types of complex misperceptions including hearing voices, feelings of a shadowy presence, of flying or being outside the body, of limb shortening/lengthening; delusions of being controlled, and hallucinations of complex visual and auditory/visual scenes involving people (to name a few). We will review these findings and link them to the schizophrenic syndrome.

**Fig. 1:**
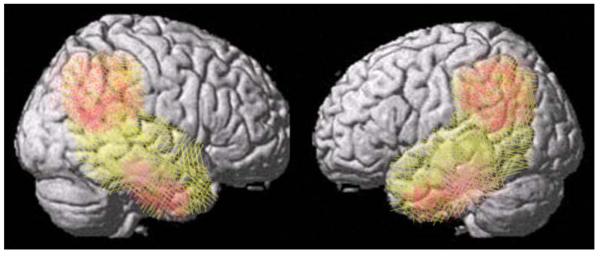
Yellow region denotes the approximate location of the key regions (shown in right and left hemispheres, respectively). This network includes the hippocampal system and medial temporal lobe, the inferior/anterior temporal lobe, superior temporal gyrus (STG) and sulcus (STS), the lateral temporal lobe, IP lobe (IP), and temporoparietal occipital junction (TPJ). Higher order unimodal and multimodal representational systems converge onto the entorhinal cortex and hippocampal system and in the posterior superior temporal and IP regions shown in red. These default mode regions are active during everyday activity. In addition, the posterior default mode structures (in red) show metabolic and structural abnormalities early in the course of Alzheimer's disease (Buckner et al. 2005). Hippocampal system activity has been shown to be correlated with activity in the inferior-lateral temporal lobe and TPJ (Kahn et al. 2008)

Considerable convergence and overlap of these functional regions can be found in healthy subjects within the lateral and posterior “default network” (approximate regions shown in red in Fig. 1), which also includes medial temporal and anterior inferior temporal regions (Gusnard et al. 2001). Activity within the TPJ (shown to be active in the “default mode”) is correlated with activity within the hippocampal system (Kahn et al. 2008). In the last part of the review, we will also discuss the ventral temporal default regions or the role of the hippocampal system in producing symptoms. The information reviewed here was previously presented in abbreviated form (Wible et al. 2007a,b, Wible 2008).

## Methods

This is not a formal meta-analysis, but a conceptually guided review and synthesis. In visual hallucinatory syndromes, the cortical modules that are involved in different component processes of vision are used. In schizophrenia, we began with a consideration of the symptoms and asked what component processes within a healthy brain could be involved. We thus based the review on conceptually framed questions about the brain bases of different functions. For example, as a first step in collecting information for the review, we started with the premise that AVH were a hallmark symptom of schizophrenia and hence reviewed regions related to language and voice processing, as well as studies of actively hallucinating schizophrenic subjects. AVH consist of both hearing a voice and of the attribution of a voice to an outside source. The conceptual component processing systems would be the system(s) used to perceive voices (e.g. What brain systems are used when hearing voices?) and to perceive that the voice is from an outside source (e.g. What brain systems are used to identify (to know) that someone in the environment is speaking?). We started researching and writing the review in September of 2006 with searches (of PubMed) articles related to speech processing and voice processing. We also searched on terms such as auditory hallucinations, hallucinations, language processing, and others. A conceptually related question would be: How are voices recognized, represented and stored? For the conceptual question of attribution and distinguishing one's own actions from those of others, we did searches from several vantage points. For example, a conceptually related question would be: What is the neural basis of the identification of one's own voice versus other voices? During hypnosis there can be a lack of attribution or awareness of actions, so we searched for articles on the brain bases of hypnosis. There is also a syndrome where the “self” is missing or people feel as though they are dead (Cotard's Syndrome); we searched for articles on the brain basis of Cotard's Syndrome. Auditory hallucinations many times have a negative emotional tone. A related conceptual question would be: What is the brain basis of negative emotions and of perceiving and producing emotional speech? As we read the papers, we also collected citations from the references and from the “related articles” function of the PubMed database. We also used Google Scholar for many of the same or similar searches done in PubMed. In the process of searching, we read (approximately) 435 papers for the review (that we had organized into approximately 80 topics/folders) and selected several to cite. For the major theoretical points, we cited papers from major research groups who have published numerous publications in highly cited journals and who have written reviews on the topics of interest. For the most part, we emphasize results that were confirmed with converging findings from several lines of research. The major theoretical points that will be made in the paper are summarized in the Tables and Figures. For the brain figures, unless otherwise noted, the regions indicated are the *peak* activations mapped onto the common space used in SPM99 and higher versions (the ICBM152 template); the actual activations are usually more extensive than indicated on the figure. Many of the figures contain activations or regions that have been taken from existing publications. We used an image editor (GIMP) to align the brains in our figures with those in the papers, but the locations depicted should be considered to be approximate. The symptoms descriptions were taken from the Scales for Positive (SAPS) and Negative (SANS) Symptoms (Andreasen 1981, 1984).

### The verbal language system

**Fig. 2:**
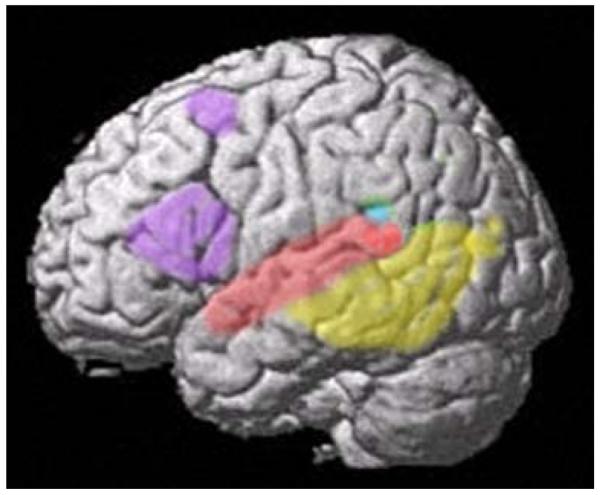
Left hemisphere language areas: yellow — interface between auditory word representations and semantic representations, pink — acoustic/phonetic speech representations, blue — area Spt for auditory-vocal interface (pre-articulatory representations) and purple — articulatory codes, modified from (Hickok and Poeppel 2004)

Theories of language processing differ in terms of the number of stages, the type of information represented in each stage, and the interactivity of the stages; see (Caramazza et al. 2000) for a review. Correspondingly, the particular functional role of each of the cortical language regions is also a matter of some debate. A typical theoretical view of speech production would start with the activation of semantic representations. The semantic representations are widely distributed in posterior cortical regions such as temporal lobe (e.g. (Tranel et al. 1997)—note that semantic representations of verbs are also housed in frontal regions). Semantic representations are then linked to auditory word forms, processing that is thought to be performed in the posterior middle temporal gyrus (Bates et al. 2003, Boatman 2004, Dronkers et al. 2004, Indefrey and Levelt 2004) — see Fig. 2. Auditory or phonological representations that are represented in the STS are then translated into motor codes for the vocal tract in the sylvian parietal-temporal area Spt (a region near the temporo-parietal boundary and usually located within the planum temporale (Hickok et al. 2000, 2002, Okada and Hickok 2006)). Area Spt is thought to interact with middle and inferior frontal regions to produce the articulatory codes for speech production. It is important to note that recent theoretical formulations posit that auditory word perception is processed bilaterally in the posterior temporal/STS regions with production, not perception, being more strongly left lateralized; see (Hickok and Poeppel 2007) for a critical discussion of language related issues. Studies of speech perception (as well as some production studies) show bilateral activation of temporal and IP regions; indicating that both hemispheres play an important role in language processing (Awad et al. 2007, Humphries et al. 2006, Ojemann 2003). It is also important to note that speech production and perception systems overlap extensively so that many of the regions used to perceive a voice are also used in thinking and speaking (e.g. STS, Spt). In the next section we will discuss the role of the IP lobe in language processing.

### The inferior parietal region — general functionality and language

The parietal region is generally thought to be involved in integrating sensory information, as well as transforming different coordinate systems into a common frame of reference in the service of action planning and in forming intentions for action (Andersen et al. 1997). The TPJ and intraparietal sulcus are thought to be responsible for combining information from several sources (e.g. visual, somatosensory, vestibular) in order to construct a moment-to-moment representation of this information; this region may also detect multi-sensory conflict within information from these different sources — see description in (Blanke and Arzy 2005). Recent studies have shown a key role for the intraparietal sulcus in at least some types of working memory (McNab and Klingberg, 2008).

**Fig. 3:**
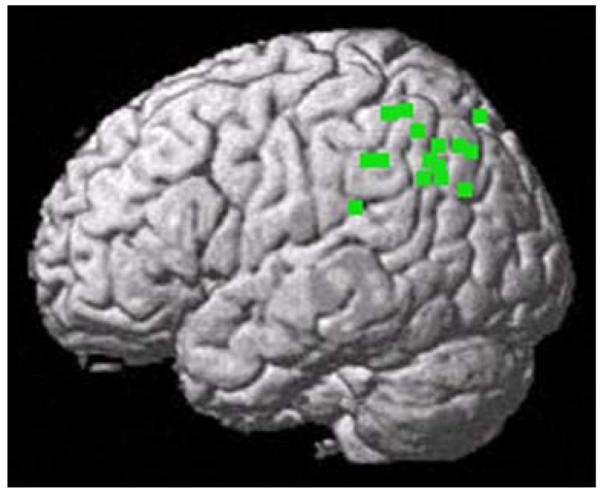
Peak coordinates (shown only for left hemisphere, but there is bilateral involvement) for activation related to phonologically coded working memory in green modified from (Jonides et al. 1998). Note also that lesions of supramarginal/angular gyrus (IP) impair verbal short term memory and that activation in IP bilaterally is related to linguistic or semantic context (e.g. Vallar et al. 1997; Humphries et al. 2006)

With regard to language processing, the IP region may participate in integrating sensory information in the service of planning verbal utterances or written language and hence is involved in short term memory processing, semantic processing and syntactic processing. Damage to the (left) IP region (areas 39/40) can result in deficits of auditory verbal short term memory (Vallar et al. 1997, Warrington et al. 1971). The IP region (bilaterally, but with some left hemisphere bias) also shows activity during verbal working memory tasks (Jonides et al. 1998); see Fig. 3. However, with regard to verbal working memory, a recent review of the neural basis of phonological working memory or short term storage emphasizes the circuits that are involved in speech perception and production, and provides new evidence from individuals with phonological working memory impairments (Buchsbaum and D'Esposito 2008). This review posits a particularly important role for the posterior superior temporal regions and especially area Spt in verbal working memory and calls for a reconceptualization of the psychological models of working memory (Buchsbaum and D'Esposito 2008).

A functional magnetic resonance imaging study (FMRI) found that the angular gyrus of the IP region was activated bilaterally during auditory sentence perception; a consistent finding in the literature (Humphries et al. 2006). The left and right IP regions were found to be associated with semantic processing, whereas only the left was also involved in syntactic processing (Humphries et al. 2006). Lesions of the IP (left angular) can also produce alexia with agraphia (inability to read and write) and sometimes pure agraphia (Goodglass et al. 1983). An analysis of the lesion site of patients with single word comprehension deficits found that lesions of the left posterior temporal and IP cortex were needed to disrupt auditory word comprehension or semantic access (Hart and Gordon 1990). Correspondingly, a separate study found that left IP damage reduced the semantic variety found in speech production (Borovsky et al. 2007). The IP region is also part of a system for integrating gestures and speech (Willems et al. 2007). Gestural production and imitation deficits are often associated with inferior parietal damage (Rothi et al. 1994). Together these studies suggest that the IP region is important for language processing (especially perception) and gestural processing.

The left hemisphere language areas shown in Fig. 2 and the IP/TPJ region, along with homologous right hemisphere regions also serve additional functional roles that may be related to other schizophrenic symptoms. As we will describe in the next sections, these areas may form a system for the analysis or processing of complex social information in the service of immediate social interaction, communication and thought.

### Progress in identifying the regions involved in producing auditory verbal hallucinations

**Fig. 4:**
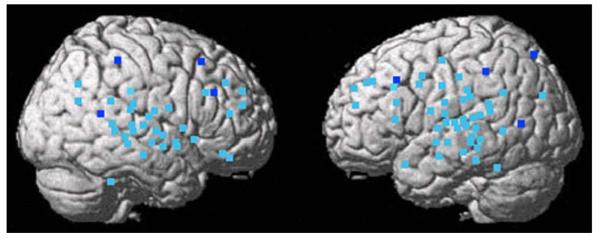
Light blue dots correspond to peak cortical activation sites for fMRI activity during active AVH in schizophrenic subjects (papers are listed in the main text). Dark blue dots are from a recent study from our laboratory (Han et al. 2007)

**Fig. 5:**
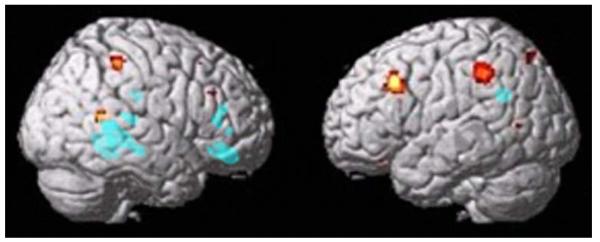
Red/Yellow: FMRI Activation correlated with AVH SAPS score during performance of an auditory word semantic priming paradigm (Han et al. 2007). Blue: FMRI activation from a single subject during hallucinations of a male voice (Lennox et al. 1999)

The large literature that has accumulated on the subject of hallucinations has been reviewed in several recent papers (Crow 2004, David 2004, Ditman and Kuperberg 2005, Seal et al. 2004, Woodruff 2004). Neuroimaging studies of actively hallucinating subjects suffering from schizophrenia have identified activity in several cortical regions. Activation of the superior temporal region (especially the right STS) is a very consistent finding; along with the bilateral IP region, the posterior middle/inferior temporal, inferior and middle frontal regions, the insula (left), the hippocampus and parahippocampal gyrus, and the cingulate (Bentaleb et al. 2002, Dierks et al. 1999, Lennox et al. 2000, Shergill et al. 2000, Silbersweig et al. 1995, van de Ven et al. 2005). Figure 4 is a summary figure of the peak cortical activation sites found in the aforementioned studies (in light blue). A recent study in our laboratory also found correlations between fMRI activity and measures of hallucinations in many of these same regions shown in dark blue in Fig. 4 — (Han et al. 2007). The results of our study were very similar to those found in a subject whose hallucinations were periodic and therefore amenable to study by fMRI; the subject heard a voice for approximately 26 sec and then no voice for approximately 26 sec (Lennox et al. 1999). In that study, a right middle temporal gyrus/STS activation preceded the hallucination and remained active throughout the experience, closely followed by activations in the right IP and inferior frontal regions (see Fig. 5 for activity related to hallucinations from (Han et al. 2007) in red/yellow and from (Lennox et al. 1999) in blue).

Measures of hallucinations also show correlations with volumetric measures of the superior temporal region and with measures of white matter in tracts connecting temporal and IP regions (Hubl et al. 2004); see (Shenton et al. 2001 for a review of volumetric studies). Together these findings suggest that the speech production/perception regions are involved in producing AVH. We also hypothesize that over-activation of semantic/syntactic processing systems within temporal and IP regions could lead to thought disorder and abnormal functioning of the IP verbal memory system could contribute to short term (working) memory abnormalities (Lee and Park 2005).

Many theoretical views assert that hallucinations are misattributions of the patient's thoughts. In addition to perceiving a voice; schizophrenic individuals misattribute the voice(s) to an outside source, the voices are often in the form of commands or comments on behavior (sometimes derogatory in nature) and some patients not only hear a single voice, but can hear conversations (David 2004). We will address these phenomena throughout the paper and will show how they may arise from abnormal activity in language related regions and surrounding cortex. We also note that activation in posterior language regions, would in most cases, be predicted to lead to obligatory activation of frontal posterior inferior and middle language regions (producing abnormal activation). We will briefly review voice processing in order to explicitly connect aspects of voice perception, representation and production found from the study of healthy individuals to AVH in the next section.

### The voice perception system

Hallucinations consist of a sensory experience in the absence of a real object. Auditory verbal hallucinations or AVH in schizophrenia consist of hearing a voice or voices (in the absence of a real voice) and of the attribution of that voice to an outside source. Each voice belongs to a person, and so voice perception not only involves phonemic and semantic processing, but also some aspects of social cognition. Emotional processing and person representation/identification are part of normal voice processing and are needed during conversation (Belin et al. 2002).

In the section on language processing, we described evidence showing that the STS represents phonological information that is used during speech perception and production. The STS is also activated by viewing facial speech gestures (Wright et al. 2003); see the blue region in the figure in Table 1. In addition, several other regions in the posterior lateral temporal cortex and IP showed fMRI activity that was related to facial speech gestures — coded in blue also in Table 1 (Calvert and Campbell 2003). It is important to note the extensive reach of regions representing facial gestures into territory that is considered to be part of a core language processing system; for example the middle temporal gyrus is thought to subserve the link between phonological and semantic representation (especially on the left — see verbal language section above). Calvert and Campbell (Calvert and Campbell 2003) propose that the left middle and posterior temporal regions are used for reading speech from faces while the corresponding regions in the right hemisphere are used for reading the identity of the person or for reading emotion. Gobbini and Haxby (Gobbini and Haxby 2006) propose that the posterior STS and TPJ are part of a system for face or person recognition. Functional imaging results correspondingly show activity in these regions that is related to familiar versus unfamiliar faces (Lotze et al. 2006); see yellow regions in Table 1.

Together these findings show that the regions that are involved in the linguistic (and especially phonological or auditory) aspects of voice perception (and production or thought) may also play a role in the representation of another person who is speaking. The facial gesture representations or person representations would be active during conversation with another person and would probably contribute to the understanding or perception that someone in the environment is speaking.

The superior temporal cortex and right IP cortex also may participate in the representation of the auditory aspects of voices in general and of familiar voices. Penfield and Perot (1963) published a comprehensive summary of the results of presurgical brain stimulation in the temporal and parietal regions. Auditory experiential responses such as hearing a voice, voices, meaningful sounds or songs were found almost exclusively after stimulation of the lateral and superior surface of the STG (see Table 1 — black numbers for the location of stimulations that produced voices). In addition, right IP and posterior temporal lesions impair the recognition of familiar voices (see Table 1 for approximate region). These regions may (at least partially) house the voice representations or voice memory of individuals; see (Belin et al. 2002) for a discussion.

In summary, the representation of auditory voice percepts and (at least part) of the representational processing that underlies the identification and perception that a person is speaking are housed within the bilateral superior temporal, STS, and right IP regions. We hypothesize that over-activation of the face/facial gesture representations during inner speech could produce the (perhaps unconscious) impression that someone else is speaking or present. Presumably overactive gesture activations could also feed forward to activate other aspects of the person identification/representation system.

As discussed briefly above, the posterior STS and TPJ regions contain tri-modal (auditory/somatosensory/visual) and bimodal representational regions that provide rapid feedback and feed-forward input to primary sensory areas and especially to auditory cortex. Multimodal influences can modulate cortical responses within very short (30 millisecond) latencies (Driver and Noesselt 2008). Thus, visual and somatosensory input are available at very early stages of auditory processing (Schroeder and Foxe 2002).

Auditory cortex may act in concert with areas of STS/TPJ in a dynamic manner such that incoming voice/phonemic information can be modulated by somatosensory and visual input, providing a way for social/gestural context to influence speech processing. Problems with this visual/somatosensory feedback to auditory cortex could contribute to misattribution errors in AVH and could also provide a mechanism for producing somatosensory and visual hallucinations. In addition, we hypothesize that over-activation of either the STG or IP / temporal lobe voice representations could result in hearing voices or in the feeling that someone is speaking.

Two patients with schizophrenia spectrum disorder who experienced AVH also described visual hallucinations of speech-like lip and mouth movements during the AVH (Hoffman and Varanko 2006). These findings are consistent with our view that abnormal activation of the STS and/or posterior middle temporal regions could contribute to the feeling that others are speaking during AVH. Abnormal agency processing could also result in a subject's inner voice being attributed to an outside source, resulting in the attribution errors seen in schizophrenic patients. The posterior STS and IP are also involved in agency processing as well as other social cognition functions (Abraham et al. 2007, Decety and Grezes 2006, Perrett et al. 1989, Saxe and Wexler 2005) and these will be discussed in the next sections.

### Social cognition and the superior temporal sulcus and inferior parietal regions

In the previous section, we described how the “voice” processing system could be related to AVH. The STS and TPJ are not only part of the neural substrate for voice processing; they also form part of a system for social cognition and agency. The majority of this research has focused on the right IP/TPJ. However, we would like to note that there is evidence that this system includes homologous left hemisphere regions to some extent — a fact that links them even more closely with certain aspects of the language system (Lloyd et al. 2006, Platek et al. 2006, Ruby and Decety 2001, Saarela et al. 2007).

The STS, ventral premotor and IP regions are components of the “mirror system”. This system is a collection of regions that are active when either observing or performing some gesture or action. The mirror system includes the STS, which then projects to the IP, which in turn sends information to the premotor cortex mirror neuron area; see (Decety and Grezes 2006) for a discussion. The IP component of the mirror system is activated in a somatotopically organized fashion during (object-related) action observation and execution in monkeys and humans (Buccino et al. 2001, Fogassi et al. 2005, Fujii et al. 2007). Abnormal functioning of the mirror system may result in reliance on stored representations rather than contextual representations in making online judgments or interpretations; resulting in some aspects of schizophrenia such as concreteness and the inability to interpret proverbs (Buccino et al. 2001).

Jardri and colleagues (Jardri et al. 2007) hypothesize that a network for self-awareness is involved in distinguishing speech production from listening to speech. Listening to speech, in healthy controls, activated the ventral premotor cortex, STS and IP (predominately on the right); all regions shown to be components of the mirror system. The production of speech (versus passive listening) de-activated the medial parietal and medial prefrontal cortices (part of a resting-state network) — as well as modulating IP activation. The authors concluded that the fact that this medial prefrontal network was modulated during self produced speech was evidence that there is a “self awareness network” that is used to generate a signal of agency or the feeling of producing speech that is also used in the resting state. In a subsequent study (Jardri et al. 2008), they extended these findings to schizophrenia. Schizophrenic subjects had increased activity in the medial fronto-parietal self-monitoring network compared to controls; along with increased activity in the right IP. The medial fronto-parietal hyperactivation was thought to result in the “excessive misattribution of their own voice to external agents”. The IP hyperactivity was also positively correlated with positive symptoms.

The TPJ or IP and STS regions, in addition to being part of the mirror system, are also heavily involved in other social cognition functions. Decety and Grezes, in an extensive review, (Decety and Grezes 2006) have designated the right TPJ as the “social brain region”; we show a modified version of a summary figure from their paper in Table 2; this figure shows that activations from theory of mind and agency studies cluster in the right TPJ. Theory of mind is the ability to attribute and represent other's mental states or beliefs and intentions or to “read their mind” (“predict the goal of the observed action and, thus, to “read” the intention of the acting individual” — from Decety and Grezes 2006). It was previously thought that the anterior cingulate or paracingulate region had played a key role in this function, but recent lesion data have challenged this view (Baird et al. 2006, Bird et al. 2004).

The STS is involved in perceiving and analyzing biological motion and goal directed behavior in the service of understanding the intentions of others and of attributing mental states to others (Abraham et al. 2007, Pelphrey et al. 2004a, b, Perrett et al. 1989, Saxe and Wexler 2005). For example, the STS, especially in the right hemisphere, was sensitive to the perceived context of shifts in gaze of a moving animated figure (Pelphrey et al. 2004b). Homologous regions in the left hemisphere showed some modulation, but much less. Gaze direction is used to indicate others' interest and focus of attention. The abnormal computation of gaze could contribute to symptoms such as delusions of reference, persecutory delusions, poor eye contact and to the social withdrawal seen in schizophrenia; see also (Hoffman 2007). Over-activation of STS gaze regions could send an unconscious signal that one is being watched and individual interpretation or reaction to this signal could result in one or a combination of these symptoms. For example, the feeling of being watched or being the focus of attention could also result in the patient concluding that they are famous or special in some way. Indirect evidence for these assertions comes from a study showing that schizophrenic subjects are more likely than control subjects to interpret averted gaze as being directed at them (Hooker and Park 2005). See Table 2 for a summary of findings related to right IP, STS, and TPJ function and schizophrenic symptoms. The reader is referred to Redcay (2008) for an extensive review of the role of the posterior STS in language and social cognition; she summarizes the function of the STS as follows:” Thus, behaviors that engage the STS include attributing intentions to others, perception of a social form from sparse, moving information, perception of the changeable aspects of faces such as eye gaze and expressions, complex motion perception, prosody perception, and narrative comprehension, to name a few.”

Together the cognitive neuroscience studies reviewed above implicate the STS and the IP region in social cognition and in the on-line computation of intent and reaction in social situations. There is overwhelming evidence that IP/TPJ regions are part of a system for the processing of agency and intention; especially in the right hemisphere — see also (Blakemore et al. 2003). Correspondingly, at least three Positron Emission Tomography (PET) studies of schizophrenia have shown that abnormal agency processing in patients is related to over-activation in (especially the right) IP or TPJ; see (Spence 2002) for a review. In healthy individuals, the feeling that someone else is acting is related to the activation of the inferior parietal region; awareness or the feeling of causing the action oneself results in the activation of the anterior insula (Farrer and Frith 2002). Healthy individuals were studied in a task where the degree of control over the movement of a virtual hand was manipulated (Farrer et al. 2003). Subjects experienced degrees of control in conditions ranging from total control (complete correspondence between virtual hand movement and the subject's movement of the hand) to a condition where it was very clear that the experimenter was moving The hand. Healthy subjects showed a relative increase in (predominately right) inferior parietal activity that was related to the feeling that someone else was controlling the hand. In other words, the less the subject felt in control of the hand, the higher the activation in the inferior parietal region. Anterior insula activation was inversely related such that anterior insula activity increased with the subject's increasing control (and increasing perception of control) over the hand (Farrer et al. 2003). A subsequent study of schizophrenia and agency used patients with Schneiderian First Rank (SFR) symptoms (Farrer et al. 2004). SFR Symptoms (Schneider 1955) consist of the belief that one's actions/thoughts are controlled/replaced/manipulated by an outside force and include voices conversing or commenting, thought broadcasting, thought withdrawal or insertion, and the belief or delusion that ones actions or thoughts are controlled by others or some outside force (sometimes also called passivity symptoms). In that study, the degree of subject's control over the movement of a virtual hand was also experimentally manipulated to varying degrees as was done in the previous study of healthy controls (Farrer et al. 2004). Schizophrenic subjects showed an aberrant relationship between their degree of control of the hand movement and the activation of the right angular gyrus (part of IP and TPJ) in conjunction with no activation of the insula. Schizophrenic subjects who had reported FRS did not show the normal modulation, or increased parietal activity with increasing lack of control over the hand. The patients only showed a relative increase in parietal activity (over the no distortion condition) in the most extreme condition where movements of the hand were actually controlled by the experimenter; in that condition there was no relationship between the patient's movement and movement of the hand. The authors summarized the findings: “In those patients for whom such modulation still occurs, activity is more likely to go above the threshold which signals that someone else is acting”. This study also showed that the right angular gyrus over-activation was positively correlated with the Schneiderian score (Farrer et al. 2004); see purple region in Table 2. Activity in the right IP (inferior and superior) was also positively correlated with the degree of Schneiderian symptoms in a PET study examining resting state activation (Franck et al. 2002). An additional study also showed that schizophrenic subjects who experienced passivity symptoms had hyper-activation of parietal (and cingulate) cortices during a motor task (Spence et al. 1997). That study employed a design where schizophrenics with passivity symptoms (or the belief that they were controlled by an outside force) were compared to deluded schizophrenic subjects who did not have passivity symptoms, to a control group, and to themselves at a later time when these symptoms abated. Schizophrenic patients with passivity symptoms (compared to healthy controls and schizophrenics without such symptoms) showed hyper-activation of inferior parietal (and cingulate) cortices. At the repeat scan session, the hyperactivation remitted in those subjects for which passivity symptoms decreased over time. The abnormal activation seen in these papers strikingly coincides with the right IP activation that is associated with imagining other's actions in studies of normal control subjects (Ruby and Decety 2001).

To summarize, the STS and TPJ/IP regions (especially in the right hemisphere) together may form part of a system for the analysis and representation of other's intentions and actions. Over-activation of the posterior STS could result in delusions of reference or the feeling that others are watching you. Over-activation of IP agency areas could contribute to a number of symptoms involving agency (see Table 2), including the attribution of ones own thoughts to an outside source, thought broadcasting, thought withdrawal and mind reading (also related to abnormal theory of mind processing). Direct evidence for over-activation of the right TPJ/IP region in schizophrenia and the relationship to symptoms was also discussed. Frith et al. (2000) have also advanced a theory of passivity symptoms. They make the association between delusions of control and the alien hand or anarchic hand syndrome (where damage to the anterior cingulate/corpus callosum/SMA causes the contralateral hand to perform actions that are unintended). Their supposition is that movements arising from external contextual cues, rather than goals, are no longer inhibited after damage to these regions. They propose that schizophrenic delusions of control occur when no predicted state (of the movement) is computed prior to a movement; or in other words, patients have an inability to form representations of the predicted consequences of actions. They mention several candidate brain regions for this mechanism, including the possibility that representations of current and predicted limb positions might also be located in parietal lobe; an assertion that would be consistent with our view. They also discuss somatosensory feedback; the patient's own sensory feedback during a movement may be amplified and thus could also contribute to feelings of being controlled. They assert that over-activity in schizophrenia results fundamentally from a lack of inhibitory signal that is associated with the prediction of stimuli or motor behaviors (possibly sent from the prefrontal or anterior cingulate regions); this is a major point of divergence between their views and the current proposal. They assert that a lack of connectivity between these inhibitory frontal regions and parietal cortex could cause delusions of control and between frontal and temporal regions could cause hallucinations or thought insertion (we believe that thought insertion stems primarily from over-activity in inferior parietal cortex).

In the next section, we will discuss how over-activation of the right STS and IP regions may also contribute to the negative emotional tone that can be found in the content of schizophrenic hallucinations and delusions.

### The right IP and STS are involved in dynamic fear processing

Within a conversation or social context, emotion perception is based on dynamic facial, body and voice intonation cues. Fear and paranoia are sometimes a part of the schizophrenic syndrome and voice hallucinations can have a negative emotional tone. Evidence is accumulating that the posterior IP and posterior STS are involved in dynamic emotional perception; especially dynamic fear perception or perception of negative emotional gestures of the body and face. These results parallel the long established findings that the right STS is involved in the perception of emotion in voices that is conveyed by prosody (Wildgruber et al. 2005).

The right IP “agency” areas that were discussed in the previous section are coincidentally adjacent to a region of IP cortex that when damaged, results in a deficit in the recognition of facial fear expressions. Thirty-seven patients with focal brain lesions distributed across the posterior cortex were asked to recognize or categorize facial expressions of happiness, surprise, fear, anger, disgust, and sadness (Adolphs et al. 1996). An analysis of lesion location and relationship to symptoms showed localized symptom-lesion correlations only for the recognition of fear and to some extent, sadness (see orange region in the brain figure in Table 3, for the location in the right IP lobe). The results of this study show that the perception of these negative emotions may be processed by a relatively localized system (perhaps in the service of reacting or escaping) and that at least one component of this system is in the IP region of the right hemisphere. As mentioned in the section on social cognition, there is evidence that IP and STS regions form a system. In an FMRI study of expressive gestures, activation of the right posterior STS was positively correlated with the perception of negative valence gestures (Lotze et al. 2006); see Table 3, red square for the peak activation site, again showing parallels between STS and IP function. A recent study of dynamic fear processing showed that FMRI activity in the posterior STS and IP was related to the perception of dynamic fearful body expressions; see Table 3, green regions (Grezes et al. 2007).

We hypothesize that elevated activity in the portions of TPJ/IP/STS responsible for fear processing could result in neutral situations being identified as emotionally negative or threatening. A study of facial emotion recognition showed that schizophrenic subjects were more likely to identify neutral facial expressions as having a negative valence (Kohler et al. 2003). In that study, schizophrenic subjects showed a reduced ability to recognize emotions, but were specifically impaired in recognition of fearful, disgusted, and neutral expressions. For a discussion of face processing and schizophrenia also see (Turetsky et al. 2007). We posit that the misidentification of neutral expressions could result from the over-activation of regions involved in representing negative valence expressions.

Over-activation of the right IP and posterior STS fear processing regions could contribute to the negative tone or negative connotation to many of the aberrant experiences felt by schizophrenic individuals and could send an unconscious signal that there is a negative social situation unfolding (contributing also to persecutory delusions and possibly delusions of reference).

Delusional jealousy is another symptom of schizophrenia that may be related to abnormal activation of the right TPJ and STS or perhaps lateral temporal regions — see (Penfield and Perot 1963). The right TPJ has been implicated in the processing of desire (Abraham et al. 2007, Saxe and Wexler 2005) and the right posterior STS is activated in relationship to jealousy in humans and monkeys (Rilling et al. 2004, Takahashi et al. 2006). The right TPJ is also activated during sexual behavior (Holstege et al. 2003).

Abnormal activity in these right or left IP and/or temporal regions may also contribute to symptoms such as flat affect. It has been suggested that schizophrenic individuals do have the subjective experience of emotion, but are much less expressive for both positive and negative emotions (Kring et al. 1993). Flat affect may stem in part from disturbances in parietal/STS systems that participate in the perception and reaction to emotional gestures or cues (or deficits in “mirroring” other's emotional states).

The stimulation of illusory emotions from temporal lobe (see discussion of Penfield and Perot (Penfield and Perot 1963) above) could also contribute to delusions of jealousy or sin/guilt, and could produce an emotional tone that would contribute to many of the paranoid/persecutory delusions.

In summary, in this section we have described a largely right lateralized system in the IP and STS for processing negative emotions in the context of dynamic social situations and have related abnormal processing in this system to symptoms having to do with negative emotional processing. Over-activation of these regions could produce the feeling that a negative or fearful social situation is unfolding or that one is being negatively judged or observed. In the next section, we will extend the discussions of agency and emotional processing. We will discuss agency processing in the context of distinguishing the self from others. We will also discuss a right lateralized posterior temporal region that may be involved in representing social scenes involving people (including negative or violent scenes).

### TPJ and posterior temporal areas are involved in representing embodiment and the body's position in space

The TPJ/IP was described above as having a role in theory of mind and agency. Representing other's intentions and emotional states necessitates a representation of the self (versus others), as does social communication. A large proportion of the work in the area of self representation has been done in Dr. Olaf Blanke's laboratory, and his group has commented on the connection between this type of processing and schizophrenic symptoms; for example, see (Arzy et al. 2006). In a recent review of their findings, they implicate the TPJ as the core region responsible for the multi-sensory integration needed to create a representation of the self and body (Blanke and Arzy 2005); see yellow region in Table 4: “The TPJ has also been involved in cognitive functions that are closely linked to self-processing and out-of-body experiences: egocentric visuo-spatial perspective taking, agency (the feeling of being the agent of one's actions and thoughts), and self-other distinction (the capacity by which one distinguishes between oneself and other conspecifics). Thus, during out of body experiences, one's visuo-spatial perspective and one's sense of agency are localized at the position of the disembodied self that is hovering above the physical body. ”

Both out of body and autoscopy experiences (seeing one's own body in extra-personal space) can be caused by damage to the TPJ or posterior temporal-occipital cortex. Six patients with these disorders were studied and the location of their brain lesions (or elicitation of the symptoms with grid stimulation of the brain) is shown in orange in Table 4 (Blanke et al. 2004). Individuals with damage to this region (it can be on either the right or left side) can have vivid or realistic feelings of floating, flying, body rotation or they may feel as though their limbs are transformed (e.g. illusory shortening) or are moving. Stimulation of the right TPJ in one neurosurgical candidate produced not only out of body experiences, but also illusory limb shortening (Blanke et al. 2002). Stimulation of a left superior temporo-parietal area in a neurosurgical patient (who did not have a psychiatric disorder) produced the feeling of a presence that shadowed changes in the patient's own position and posture (Arzy et al. 2006). The patient not only felt this presence during brain stimulation, but also attributed motives to the illusory figure “He wants to take the card”. “He doesn't want me to read”. The stimulation sites are shown in Table 4 in green. Symptoms such as some somatic hallucinations, somatic delusions, and persecutory delusions (e.g. the feeling of being followed) could conceivably follow abnormal activation of these functional regions in the TPJ and posterior temporal/occipital lobe. Abnormal activation in this region could produce feelings of flying or out of body experiences that could be misinterpreted with religious connotations, resulting in other delusional symptoms. As reviewed in (Decety and Lamm 2007), damage to the right TPJ can also produce delusional beliefs about the body or somatoparaphrenia in individuals who did not have schizophrenia as well as a lack of awareness of the condition of the body (asomatognosia).

Additional evidence that this area is important for schizophrenia comes from a high-resolution EEG study of mental imagery of the subject's own body. This study showed that prolonged right temporo-parietal activation was associated with disturbances in self and body processing and with schizotypy scores (Arzy et al. 2007).

### TPJ and the posterior temporal lobe are involved in the representation of social scenes involving people

The posterior temporal region may also have a role in storing memory for and in processing complex scenes involving people. Stimulation of the lateral posterior right temporal region produced complex visual experiences of persons, scenes, group of peoples, or recognizable objects (only a few stimulation sites on the left produced these experiences). Plotted in Table 4 (red numbers) are responses involving visual hallucinations of complex scenes. Action involving people was most frequently reported from these stimulations. These regions are also present in the planum temporale of both hemispheres (not shown in Table 4). Penfield and Perot noted that the right hemisphere experiential responses were in areas that would have been part of the language system in the left hemisphere. Note also that there have been several recent reports of an extrastriate body representation area in posterior temporal/occipital cortex and also of a representational system for body movement in the ventral frontal cortex (David et al. 2007, Pourtois et al. 2007, Urgesi et al. 2007). See also the discussion in the previous section of fearful body expression (Grezes et al. 2007).

Over-activation of these regions in the posterior temporal/occipital lobe could produce a conscious hallucination of a social scene involving people (one of the symptoms of schizophrenia). Over-activation could also cause an unconscious feeling that one is in a social situation or a negative social situation; contributing to paranoia and other delusions and symptoms, including persecutory delusions and delusions of conspiracy or feelings of being followed (see Table 4).

### The hippocampal system/ ventral and medial anterior temporal regions and schizophrenia

The hippocampus and surrounding cortex are functionally and anatomically interconnected with the regions discussed thus far in our review and have been shown to be abnormal in schizophrenia (Coyle 2004, Heckers 2001, Heckers and Konradi 2002). By virtue of intimate connections with the higher order representational areas discussed above, and a propensity for over-activation, we believe that the hippocampus is involved in the production of schizophrenic symptoms in a specific manner (Wible et al. 1997). Hippocampal system activity is correlated with TPJ and with lateral anterior temporal lobe activity; and activity in these regions predicts recollective success, as well as being part of the posterior default network (Stark and Squire 2001, Vincent et al. 2006). A recent study examined detailed profiles of FMRI connectivity within subregions of the hippocampal system using data from 100 subjects (Kahn et al. 2008). The results revealed two pathways whose activity was correlated with distinct sub-regions within the hippocampal system; activity in the body of the hippocampus and the posterior parahippocampal gyrus was correlated with lateral parietal cortex and activity in the anterior hippocampus-perirhinal/entorhinal regions was correlated with lateral anterior and temporal pole regions. Functional connectivity is not well understood at a neural level and the consequences of the detection of these networks have not been fully worked out. Hence, any links between default network or hippocampal network regions and pathological mechanisms should be considered to be highly speculative. However, given the results of the Kahn et al. study cited above; overactivation of the hippocampus could not only activate the TPJ, but could potentiate this region over time.

The hippocampus is prone to over-activation, is the most frequent site of epileptic foci, of damage after anoxia or ischemia, and contains the highest concentration of glucocorticoid (stress hormone) receptors in the brain; and contains one of the highest concentrations of (N-methyl-D-aspartate (NMDA) receptors (a type of glutamate receptor) in the brain (Cotman 1987, Sapolsky 1994). The NMDA receptor is thought to play a critical role in synaptic plasticity or long-term potentiation. As discussed in previous sections, the TPJ has also been shown to contain bi-modal and tri-modal representational systems that could provide rapid feedback to primary cortical regions. Over-activation of the TPJ could also produce activation of auditory, tactile and visual regions; providing an additional mechanism for creating some symptoms, especially hallucinations.

Hippocampal interaction with the TPJ could produce psychosis — when epileptics underwent temporal lobectomy (unilateral) and also had a (previously undetected) seizure focus in the remaining hippocampus, psychosis was produced as a result of the temporal lobectomy — and this process evolved over time (Levine and Finklestein 1982). Conversely, temporal lobectomy (resection of the hippocampus — and presumably removal of the focus of over-activation) resulted in a recovery from psychosis in a patient with temporal lobe epilepsy (Marchetti et al. 2001).

Hence, we hypothesize that over-activation of the regions reviewed above could stem, at least in some cases, from the hippocampus. Hippocampal damage was found to result in AVH in a non-psychotic individual (Suzuki et al. 2003). Visual hallucinations were associated with hippocampal activity in schizophrenic subjects (Oertel et al. 2007). Patients with temporal lobe epilepsy (with a focus in hippocampus) can also present with a syndrome of hyper-religiosity and/or with olfactory hallucinations. The entorhinal cortex, the major output/input region of the hippocampus, contains an olfactory region and is intimately connected with olfactory cortex. These considerations are examples of how over-activation of the hippocampus functionally related cortical regions could contribute to the schizophrenic syndrome. Table 5 briefly summarizes some points regarding hippocampal system involvement in schizophrenia and we will discuss this system in a subsequent paper.

## Conclusions

### A system of anatomically related structures

The regions reviewed here also have special functional and developmental characteristics that may provide clues to their involvement in schizophrenia. As discussed above, the TPJ is part of the default network — a system of brain regions that are active much of the time in normal individuals (Vincent et al., 2006). Activity in the TPJ region is also associated with consciousness; the activity of this region shows a relative increase upon recovery from coma, and in wakeful versus sleep states; see Fig. 3, page 691 from (Gusnard et al. 2001). Major portions of this network have been shown to be those regions that accumulate amyloid deposits in Alzheimer's Disease; however, only the posterior portions of the system, including the lateral temporoparietal and medial temporal regions emphasized in this review, exhibit abnormal metabolic activity and atrophy (Buckner et al. 2005). We postulate that the metabolic demands of constant activity in this circuit could contribute to abnormalities of this system in schizophrenia.

A number of mechanisms that control cortical excitability could result in over-activation of these systems that, over time, would also result in degeneration. We have noted in the course of reviewing findings from the literature that functional and structural abnormalities are often found within a tightly neuroanatomically interconnected set of regions related to the hippocampus and TPJ (entorhinal and parahippocampal cortices, STS, STG, amygdala, cingulate, orbital and inferior frontal and insular regions). The cingulate, orbital frontal, inferior frontal and insular regions are primary afferent regions to the medial temporal, temporal and IP/TPJ areas reviewed here.

Several candidates for the cellular abnormalities in schizophrenia have been offered that may result in over-activation of cortical regions, including ones involving altered dopaminergic or glutamatergic neurotransmission, or an altered interaction of the two neurotransmitter systems (Javitt 2007, Rujescu et al. 2006). In addition, any factor effecting the so-called up and down states or cortical excitability within the default network (including dopaminergic modulation) could lead to activational abnormalities; see (Haider et al. 2006, Vincent et al. 2006) for descriptions of up-down states. Geschwind (Geschwind 1965) noted that the IP lobule (angular and supramarginal gyri) is one of the last regions in the brain to be myelinated. Correspondingly, schizophrenia has an onset in late adolescence. A recent study examined the developmental course of discourse processing and found dramatic changes from middle childhood to adolescence (Dick and Small 2008). For example, the right posterior superior temporal region was shown to undergo changes in both the magnitude of functional activity and in connectivity (with inferior parietal regions on the right) throughout this period.

The fact that schizophrenic individuals each show a unique constellation of symptoms may stem from slight variations in the brain abnormalities within the broad system outlined in this review. Some findings of hypofrontality or abnormal frontal activation in patients may stem from abnormal IP functioning as a link between temporal and frontal regions. The exact nature and location of many of the components of the systems discussed in this review are only just beginning to be understood. Within this paper, we provide a framework for viewing schizophrenic symptoms and some suggestions for specific symptom/function/region associations. Progress in understanding the functional subdivisions and processing characteristics will lead to more accurate predictions of how and where schizophrenic symptoms arise; and also to a deeper understanding of the mechanisms involved.

### The relationship of the current framework to other theoretical views of schizophrenia

The idea that temporal lobe over-activation plays a role in schizophrenia has had a long history (Fletcher et al. 1998, Frith et al. 1995, Hazlett et al. 2000, Hubl et al. 2004, Kubicki et al. 2003, O'Leary et al. 1996, Yurgelun-Todd et al. 1996); see (Gur 1978) for a review. Trimble (Trimble 1991) and many other researchers have also noted a relationship between hippocampal abnormalities and schizophrenia.

The links between abnormalities in social processing systems and schizophrenia have been discussed in detail within a review of neuroimaging findings related to this topic (Brunet-Gouet and Decety 2006). The authors hypothesize that the medial prefrontal cortex, the amygdala, and the IP lobule are involved in producing the social cognitive deficits seen in schizophrenia and they link dysfunction in these regions to a physiological model of schizophrenia. These authors focus on executive functioning and inhibition, emphasizing cingulate and frontal contributions to theory of mind, modulation of emotion, and schizophrenia. They theorize that abnormal dopamine modulation, particularly of the anterior cingulate region, results in an inability to maintain a stable and relevant social and emotional context for others' and ones own behavior. They also present specific hypotheses; for example, that persecutory delusions could result from an abnormal evaluation of social threat. They do link abnormal agency processing to inferior parietal lobe function, but believe that there is a complicated relationship between agency and mental state representation that forms the basis for misattributions in schizophrenia. Their approach and theoretical views differ substantially from those presented in this paper; especially regarding the role of the anterior cingulate or frontal regions in producing symptoms. Our view is that most symptoms are a result of over-activation of the posterior representational systems. We believe that the anterior cingulate may be involved in individuals who exhibit abnormal collecting behavior (Anderson et al. 2005).

There are other theoretical views that are consistent or partially consistent with those reported here. Hoffman (2007) hypothesizes that schizophrenia results from a deafferentation of the social brain regions following early social withdrawal and his paper notes many correspondences between schizophrenic symptoms and social processing, an aspect that is consistent with our framework. A recent meta-analysis of neuroimaging results within the TPJ (Decety and Lamm 2007) reported that this region is involved in “…comparing signals arising from self-produced actions with signals from the environment”. This theoretical view is very consistent with proposals that some symptoms (delusions of control) stem a deficiency in self monitoring or in a deficit in the awareness of the predicted sensory consequences of their own actions (Frith et al. 1992, 2000). Finally, after an initial peer review had been completed of our paper, we found a paper which reviews the possible role of the IP region in schizophrenia, and provides complimentary information about the link between schizophrenia and this region (Torrey 2007).

Most of the prominent views of schizophrenia postulate frontal lobe involvement in the production of the symptoms through abnormalities in executive control, inhibition and working memory. Abnormalities in attention, inhibitory control, corollary discharge, the internal monitoring or initiation of willed actions and frontal-temporal connectivity have also been hypothesized (Carter et al. 1998, Cohen and Servan-Schreiber 1992, Frith et al. 1995, Goldman-Rakic 1991, Heinks-Maldonado et al. 2007, Honey and Fletcher 2006, Weinberger 1993). Schizophrenia has also been described as a syndrome involving large scale abnormalities of synaptic efficacy, especially within systems responsible for emotional learning and memory (Friston 1998). One theory posits that schizophrenia is a “misconnection” syndrome. In this view, the coordination of activation via the cortico-cerebellar-thalamic circuit; a circuit that may normally coordinate mental and motor activity, is hypothesized to be abnormal and to produce a “cognitive dysmetria” (Andreasen 1999). For this view, thought disorder is the defining symptom of schizophrenia.

In the interest of parsimony, the central theme of our framework is that the symptoms of schizophrenia can be accounted for without necessarily evoking abnormalities in a central control mechanism or in large scale coordination of brain systems. However, our framework is still partially consistent with many of these types of theories. For example, if abnormal corollary discharge produces over-activation during inner speech, perhaps our framework can be used to expand the list of symptoms that can be accounted for by the corollary discharge theory. It is also possible that corollary discharge is sent from IP (or insular) regions to the temporal lobe, resulting in symptoms. We would like to note that even if these theories are correct, they must still somehow account for the specific symptoms of schizophrenia, or in other words, why the control mechanisms, plasticity, or coordination fails for some functions and not others.

As well as describing plausible neural substrates and links to processing systems for many symptoms, we believe that our framework accounts for the fact that AVH and language symptoms, as well as verbal memory deficits, are prominent in the syndrome. We hypothesize that a major function of the system outlined in this review is in verbal communication and verbal memory. Over-activation of this system also accounts for the way in which schizophrenic subjects interpret neutral stimuli. The fact that fear or negative emotion expressions are processed within a right posterior temporal/inferior parietal system could also account for the negative emotional tone seen in some patients. Other emotions do not seem to have as much as a localized system within posterior cortex (Adolphs et al. 1996). The TPJ has also been implicated in studies that require attention or re-orienting (Decety and Lamm 2007) and the event-related potential (ERP) component, the P300 is thought to index this processes, is related to activation of the TPJ, and is one of the most consistently reported ERP abnormalities in schizophrenia (Turetsky et al. 2007).

### Verifying the assertions of the language corridor framework

Abnormalities of these systems may be difficult to detect in conventional neuroimaging experiments because the anatomical structures described in our framework are active during baseline conditions. We generally expect that symptom related activity would be higher during rest or baseline than during the performance of specific tasks. Hence, elevated baseline activity within these regions should be particularly related to symptoms. Of course, symptoms could obviously occur any time within an imaging session and some individuals may have the experience of very frequent or constant symptoms. It is important to note that studies of non-psychotic individuals with visual hallucinations can show tonic as well as episodic elevations in activity within the visual cortical modules related to producing the particular type of visual hallucinations (Santhouse et al. 2000). Studies linking resting state and baseline analyses to detailed clinical information with large numbers of subjects and/or morphometric studies with large numbers may be able to detect functional abnormalities that are correlated with symptoms. It is important to note that if activity (especially in the baseline) is elevated in the regions that we have discussed, then this will affect task related activity in these regions.

It is also important to recognize that if the representations in temporal and TPJ lobe are abnormally activated, then the efferent regions of the medial and lateral frontal lobe (including the language regions), would also be likely to show abnormal activity. We would predict that structural abnormalities would initially be mostly present in the core posterior regions reviewed here, and then over time abnormalities would spread to primary and then secondary efferent structures.

### Summary

A framework for understanding schizophrenic symptoms was presented; it is claimed that the particular symptoms that make up the schizophrenic syndrome arise from over-activation within a brain system that includes the TPJ, the hippocampus, and closely associated brain regions. It is assumed that hallucinations stem from over-activation (activation in the absence of a real object) and hence, for the sake of parsimony, we consider how over-activation could produce the other symptoms of schizophrenia. It is possible that overactivation could also result in damage and hypo-activation of these circuits in chronic patients with persistent symptoms. We realize that it is also possible that some symptoms (e.g. unchanging facial expression, decreased spontaneous movements, paucity of expressive gestures, poor eye contact, lack of affective response, lack of vocal inflections and alogia) could be a result of lack of activation, or hypo-activation of the circuits discussed. However, again, in the interest of parsimony, we are positing that over-activation underlies all symptoms and will use this hypothesis as a starting point.

The activity of the TPJ is correlated (shown in functional imaging connectivity studies) with hippocampal system activity. Hence we also assume that abnormal activity within the hippocampus and related structures could also affect activity within the TPJ. The posterior STS and IP areas (within the TPJ) contain bi-modal and tri-modal representational regions that may provide rapid feedback and feed-forward influences on the ongoing activity within unimodal cortices (and especially auditory cortex). Somatosensory representations, auditory speech representations, visual representations of people and scenes, are evident within the territory of the TPJ; providing the opportunity for these representational systems to be aberrantly activated by the tri- and bi-modal regions. This could provide at least part of the mechanism for producing auditory, somatic, and visual hallucinations in schizophrenia. The TPJ has also been implicated in attention and hence abnormal functioning could result in symptoms associated with attentional problems. The STS contains speech and gesture representations that are used during dynamic social, emotional and communication functions. Abnormal activation of these representational systems could cause symptoms related to hallucinations as well as abnormal social and emotional functioning. Symptoms associated with abnormal expressive gestures (or the perception of gestures in others) such as poor eye contact, lack of affective response and vocal inflection, unchanging or paucity of expressive gestures, inappropriate facial gestures and delusions of reference are hypothesized to be especially linked to abnormal activity within the posterior STS. The role of the inferior parietal region in planning actions, agency, self representation, and theory of mind leads us to posit that symptoms involving the abnormal computation of agency (delusions of being controlled, mind reading, thought broadcasting and withdrawal), some aspects of bizarre behavior, apathy, avolition and unusual ideas about other's intentions could stem from abnormal activity in this region. The relationship of the (right) TPJ to dynamic fear perception and intimate aspects of social behavior links this region to symptoms having to do with those functions such as flat affect, delusional jealousy, and symptoms that may be related to (unjustified) feelings of being within a fearful or negative social situation. The TPJ is thought to be involved in the semantic and syntactic aspects of linguistic function and hence abnormal function in this region could possibly produce some forms of thought disorder (see above section on language and inferior parietal lobe). Over-activation of the anterior and inferior/lateral temporal regions may also play a role in producing thought disorder because of their involvement in semantic representation (Patterson et al. 2007); note that these regions also show high connectivity with hippocampal system activity.

At a more abstract level of analysis, TPJ function may be characterized as the moment-to-moment multimodal integration of information in the service of the perception and production of contextually dependant verbal and behavioral responses (especially within social and communicative situations). In this role, the region may coordinate the rapid access of information representing the emotional and personal characteristics of individuals (for speech reaction/interpretation) and of semantic representations (for speech perception/production). We believe that abnormal functioning of this system can lead to the symptoms of schizophrenia.

## Figures and Tables

**Table 1:** Voice Perception System.

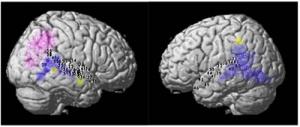	
Activation is Related to:	STS is activated by facial speech gestures and voices – indicated roughly in **blue** (Lotze et al., 2006; Wright et al., 2003). The presentation of facial movement during speech without sound activated TPJ and posterior lateral temporal regions shown also in **blue** (Calvert & Campbell, 2003). The STS is activated by phonological information during speech production and perception (see (Hickok & Poeppel, 2007); figures 3 and 4). The presentation of familiar versus unfamiliar faces – area IP and right STS **yellow** (Platek et al., 2006).
Stimulation or Lesion Produces:	Stimulation of STG bilaterally: Stimulation points that produced hearing a voice or voices (numbers in figure), modified from (Penfield & Perot, 1963). Examples are (12) voices along a familiar stream; (13) voices coming from familiar buildings; and (21) patient's cousins laughing. Lesions of right IP and temporal lobe impair the recognition of **familiar** **voices** – approximate region indicated in **purple** (e.g. (Van Lancker *et al.*, 1988); see (Belin et al., 2002) for a discussion). Lesion of Left IP: Conduction aphasia (perhaps due to area Spt), difficulties with temporal sequencing, perseveration, apraxia (difficulty in sequencing behavior.), short-term verbal working memory deficits; anomia, agraphia, alexia.
Relevant Findings	Stimulation of left IP produces thought disorder in sign language (Corina *et al.*, 1999). Actively hallucinating schizophrenic subjects show activation of the superior temporal region during the auditory hallucination.
Over- activation May Produce:	Activation of voice representations and voice memories. Feeling that someone is present and that someone else is speaking. Disturbances in the temporal sequencing and contextual processing of language and motor (gestural) behavior. Over-activation of short-term verbal memory or internal thought. Perseveration and lack of awareness of behavior.
Symptom (s)	**Auditory Hallucinations** Abnormalities in facial gesturing (unchanging expression, paucity, poor eye contact, lack of affective response and of vocal inflection and inappropriate gestural responses). Thought Disorder and Alogia Bizarre Behavior Avolition and Attention

**Table 2:** 

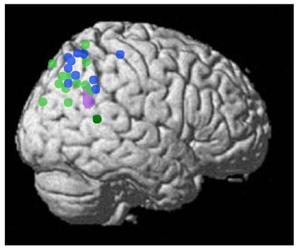	
Summary	Agency, Theory of Mind, and Intention
Activation is Related to:	Agency (**blue**) and theory of mind (**light green**) – summary figure adapted from (Decety & Grezes, 2006). Peak STS activity (**dark** **green**) related to the observation of biological motion in the service of analyzing intention(Pelphrey et al., 2004a). Activity also related to direction of gaze (Pelphrey et al., 2004b).
Stimulation or Lesion Produces:	Belief that body is controlled by external forces, feeling of no thoughts (Mesulam, 1981). Damage to right TPJ produces psychotic symptoms (Levine and Finkelstein, 1982).
Relevant Findings	Over-activation in schizophrenics (**purple**) correlated with levels of symptoms involving abnormal agency attribution (Farrer et al., 2004).
Over-activation May Produce:	Misperceptions of agency for thoughts and movements. Feeling that someone else is acting or that actions are controlled by someone else. Feelings that thoughts and actions belong to someone else. Feeling of having no thoughts. Dysfunction of the system related to computing intentions of others; incorrect attribution of intentions and the significance of other's actions. Lack of insula activation could produce poverty of speech
Symptom (s)	Delusions of Being Controlled Thought Broadcasting, Insertion, Withdrawal Delusions of Mind Reading Bizarre Behavior - Social and Asociality Delusions of Reference Attentional Deficits Some types of Persecutory Delusions Poor Eye Contact

**Table 3:** 

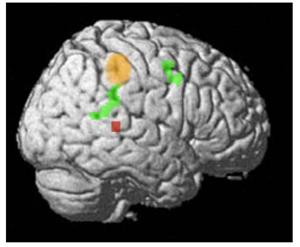	
Summary	Perception of negative expressions.
Activation is Related to:	Negative valence expressions (red) in Right STS.(Lotze et al., 2006). Mirror system, intention, perception of emotional gestures (Buccino et al., 2001; Pelphrey et al., 2004a; Pelphrey *et al.*, 2003). Activation of the right posterior temporal TPJ region is related to perceiving fear from body expressions – shown in green (Grezes et al., in press). Activation of the right posterior STS is also related to jealousy (Rilling et al., 2004; Takahashi et al., 2006).
Stimulation or Lesion Produces:	Lesion (**orange**): Inability to recognize negative valence expressions (Adolphs et al., 1996)
Relevant Findings	Schizophrenics are impaired in recognition of negative valence expressions (Kohler et al., 2003).
Over-activation May Produce:	Feeling of negative emotions, feeling of being in a negative/fearful social situation. Feeling surrounded by fear.
Symptom (s)	Flat Affect Negative tone of delusions and hallucinations Bizarre Behavior – Aggressive Persecutory Delusions Delusional Jealousy Social Withdrawal – Asociality Visual Hallucinations of People

**Table 4:** 

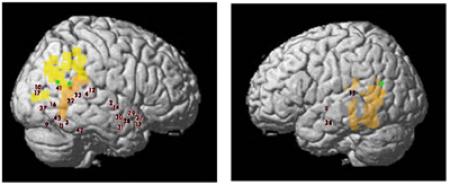		
Summary	Largely in the Right Hemisphere: Visual social scene, self/body.	Left hemisphere: Self and body processing with some visual percepts.
Stimulation or Lesion Produces:	Stimulation (**red numbers**): People acting (e.g. (8) a familiar man grabbing a stick; (15) a man fighting; (37) a familiar menacing man; (41) men with guns) – modified from Penfield and Perot (Penfield & Perot, 1963). Stimulation (**green**) produced out of body (feelings of flying), illusory limb shortening, feeling of a shadowy presence (Blanke et al., 2002). Lesion or damage in IP can produce Cotard Delusions – relevant to issues regarding self processing and somatic delusions (Gardner-Thorpe & Pearn, 2004).	Bilateral and Left Hemisphere: Lesion (**orange**): Out of body, autoscopy, feeling of flying, limbs altered, modified from (Blanke et al., 2004) Stimulation (**green**): feeling of being followed, modified from(Arzy et al., 2006)
Relevant Findings	**Yellow** region is thought to be the multimodal convergence region for representation of the self within the context of the body, extrapersonal space, and self/other perception (Blanke & Arzy, 2005). Prolonged right temporo-parietal activation (time window 310-390 ms) was positively associated with disturbances in self and body processing and schizotypy scores.(Arzy et al., 2007)	
Over- activation May Produce:	Feelings that there are people surrounding you, feelings that there are complex social situations unfolding. Feelings of flying, limb shortening/lengthening.	Feelings of flying, limb shortening/lengthening, or of being followed.
Symptom (s)	Visual Hallucinations – Figures of People Persecutory Delusions - Conspiracy Persecutory Delusions - Being Followed Somatic Hallucinations - Body Changed Shape/Size Somatic Delusions Religious delusions	All of the symptoms predicted for the right hemisphere and especially feelings of being followed. Less frequently visual hallucinations.

**Table 5:** Hippocampal system: hippocampus and surrounding cortex in the ventral temporal lobe

Activation is related to:	Activated by higher order cortical representations that are processed in an ongoing fashion
Stimulation or lesion produces:	Lesion produces profound deficits in anterograde memory. The hippocampus is interconnected to higher order cortical regions that represent the elements of perception and language
	A partial lesion produced auditory verbal hallucinations of a derogatory nature in one case(Suzuki et al. 2003)
Relevant findings	Over-activation during epileptic seizure often produces olfactory hallucinations and hyper-religiosity (e.g. (M. Trimble and Freeman 2006, Wuerfel et al. 2004)), possibly due to the hippocampal over-activation of immediately surrounding cortex. Hippocampal resection ameliorated psychotic symptoms (Marchetti et al. 2001)
Over-activation may produce:	Over-activation could result in the stimulation of any surrounding multi-modal or higher order unimodal region
	Semantic and higher order representations are stored and accessed in surrounding cortex; over-activation might produce semantic difficulties and interpretive difficulties
Symptom (s)	Olfactory hallucinations
	Religious delusions
	Grandiose delusions
	Delusions of sin
	Thought disorder
	Auditory hallucinations
	Could theoretically produce any of the symptoms because of interconnectivity with TPJ/STS/STG and amygdala

## Appendix 1

Short Description of Symptoms Paraphrased/Summarized from Symptoms of Schizophrenia from the Scale for the Assessment of Positive (SAPS) and Negative (SANS) Symptoms.

Positive Symptoms:

Hallucinations:

Auditory Hallucinations — Most common is hearing voices and the voices are typically unpleasant and negative.

Somatic Hallucinations — Can include burning sensations, tingling, and perceptions that the body has changed (shape/size).

Olfactory Hallucinations — Typically unpleasant smells.

Visual Hallucinations — Typically present as figures of people or human-like forms and can be religious characters.

Delusions:

Persecutory Delusions — Individuals believe that there is a conspiracy against them or that they are being persecuted and commonly believe that they are being followed, bugged, that mail is opened, the telephone is tapped or that someone is harassing them. Some delusions are well formed and elaborate (e.g. the government believes that they are a secret agent).

Delusions of Jealousy — The individual believes that their mate is having an affair with someone.

Delusions of Sin or Guilt — The individual feels as though they have done something terrible, or caused a catastrophic event and may feel as though they should be punished by society.

Grandiose Delusions — The individual believes that he has special powers, abilities, is famous, or is creating some special work and can be suspicious that others are trying to steal their ideas.

Religious Delusions —The individual has false beliefs of a religious nature.

Somatic Delusions — The individual believes that their body is abnormal or changed (rotting, enlarged, unusual facial features).

Ideas and Delusions of Reference — The individual believes that remarks, statements, or events refer to them, or that people are watching and/or talking about them. Shows or articles are talking about them or have some special meaning for them.

Delusions of Being Controlled — The individual thinks that their feelings or actions are controlled by some outside force.

Delusions of Mind Reading — The individual thinks that people can read his mind or know his thoughts.

Thought Broadcasting — The individual believes that his thoughts are broadcast (sometimes heard by others) and sometimes experiences their own thoughts as a voice outside the head.

Thought Withdrawal —The individual believes that their thoughts have been taken out of or removed from their mind.

Bizarre Behavior:

There are several subcategories that are usually scored for this symptom. Bizarre behavior may consist of wearing inappropriate or strange clothing or changing one's appearance. Social and sexual behavioral are sometimes inappropriate or strange (inappropriate sexual displays/gestures, muttering talking to strangers about one's personal life, suddenly start praying on knees). Behavior may also be aggressive and threatening or repetitive or stereotyped.

Positive Formal Thought Disorder

There are several subcategories of thought disorder. Derailment or loose associations, tangential speech, incoherence or word salad (as in posterior aphasia), illogicality, circumstantiality, pressure of speech, distractible speech and clanging.

Negative Symptoms:

Affective Flattening or Blunting

There are several aspects to this symptom category; these include unchanging facial expression, decreased spontaneous movements, paucity of expressive gestures, poor eye contact, lack of affective response, lack of vocal inflections and inappropriate affect (e.g. smiling inappropriately).

Alogia

Aspects of impoverished thinking, including poverty of speech and content of speech, the interruption of speech (blocking), slowed speech reaction.

Avolition — Apathy

This symptom category can be evident in a number of areas; grooming/hygiene, impersistence or inability to keep a job or in school, and physical anergia (inactivity).

Anhedonia — Asociality

The person may have no or few social interests or hobbies, decreased sexual activity and interest, inability to form intimate relationships with people, and have few friends.

Attention

Attention can be impaired with respect to social activities or situations and/or during testing.

## Appendix 2

Theory of Mind: is the ability to attribute mental states—beliefs, intents, desires, pretending, knowledge, etc.—to oneself and others and to understand that others have beliefs, desires and intentions that are different from one's own (from Wikipedia – Premack, D. G. & Woodruff, G. (1978). Does the chimpanzee have a theory of mind? *Behavioral and Brain Sciences*, *1*, 515–526).

Agency: “…the feeling of being the cause of one's own actions, desires, or thoughts, which relies on the comparison between self generated and externally produced sensory signals…” from (Decety and Lamm, 2007).

The Hippocampal System and Default Mode: Connectivity analyses use data from FMRI procedures and correlate the signal of one structure or region with the signal from other regions of the brain. This type of analysis has been used to identify regions or systems whose activity is correlated under certain conditions. Kahn et al., (2008) show that the hippocampus and closely related anatomical structures have activity that is correlated with different parts of cortex. Anterior temporal lobe activity was specifically correlated with activity in perirhinal and entorhinal regions. Medial cortex (both in anterior and posterior regions) and the temporal parietal junction activity was specifically correlated with activity in the body of the hippocampus and with the parahippocampal gyrus.

Out of body — a feeling of being outside one's own body.

Autoscopy — seeing one's own body in extrapersonal space.

Somatoparaphrenia — delusional beliefs about the body.

Asomatognosia — lack of awareness or insight about the condition of the body.

## Abbreviations

FMRI: Functional magnetic resonance imaging
IP: Inferior parietal
PET: Positron emission tomography
ERP: Event-related Potential
STG: Superior temporal gyrus
STS: Superior temporal sulcus
TPJ: Temporal-parietal occipital junction
